# Barriers Perceived by Managers and Clinical Professionals Related to the Implementation of Clinical Practice Guidelines for Breastfeeding through the Best Practice Spotlight Organization Program

**DOI:** 10.3390/ijerph17176248

**Published:** 2020-08-27

**Authors:** Antonio Jesús Ramos-Morcillo, David Harillo-Acevedo, David Armero-Barranco, César Leal-Costa, José Enrique Moral-García, María Ruzafa-Martínez

**Affiliations:** 1 Faculty of Nursing, University of Murcia, 30100 Murcia, Spain; ajramos@um.es (A.J.R.-M.); maruzafa@um.es (M.R.-M.); 2 Maternal-Pediatric Unit, Hospital Rafael Mendez, 30813 Murcia, Spain; 3 Physical Activity and Sports Sciences, Faculty of Education, Pontifical University of Salamanca, 37007 Salamanca, Spain; jemoralga@upsa.es

**Keywords:** barriers, guideline, breastfeeding, clinical practice guideline, BPSO, nursing, science implementation, midwifery

## Abstract

International institutions facilitate the contact of health professionals to evidence-based recommendations for promoting exclusive breast feeding (BF). However, the achievement of good rates of exclusive BF is still far from the optimum. The intention of the present work is to determine the barriers identified by managers and health professionals involved in the implementation and sustainability of Clinical Practice Guidelines (CPG) for breastfeeding under the auspices of the Best Practice Spotlight Organization program. A qualitative research study was carried out. The participants were managers, healthcare assistants, nurses, midwives, pediatricians and gynecologists. Semi-structured interviews were conducted which were transcribed and analyzed using the six steps of thematic analysis. Twenty interviews were conducted, which defined four major themes: (1) Lack of resources and their adaptation; (2) Where, Who and How; (3) Dissemination and reach of the project to the professionals; and (4) The mother and her surroundings. This research identifies the barriers perceived by the health professionals involved in the implementation, with the addition of the managers as well. Novel barriers appeared such as the ambivalent role of the midwives and the fact that this CPG is about promoting health. The efforts for promoting the implementation program should be continuous, and the services should be extended to primary care.

## 1. Introduction

The interest in the application of recommendations for promoting breastfeeding (BF) in clinical contexts appeared in the 1990s [1]. Since 1991, with the Baby-Friendly Hospital Initiative (BFHI), the WHO and the UNICEF have made contributions to motivate health organizations in the world to support BF through the implementation of 10 steps for successful BF [2]. More recently, public organizations and professional associations have also developed clinical practice guidelines (CPGs) for facilitating the contact of healthcare professionals to recommendations based on current scientific knowledge for promoting BF [3,4]. Even then, at the worldwide level, the 50% rates of exclusive BF are far from being achieved at 6 months after the birth of the infant, an objective set out by the WHO for 2025, and the implementation of these recommendations has been inconsistent according to countries and even regions [2].

Starting with programs such as the BFHI or the implementation of CPGs for BF at the level of health organizations implies a complex process of change, since obtaining good results depends on multiple factors and is influenced by different types of barriers [5,6]. Knowledge about these barriers could help in the creation and implementation of guides, the design of effective interventions, and the planning of strategies that are more adequate for facilitating their use [7,8]. Research on CPG implementation barriers is highly documented. A systematic meta-review which included 25 systematic reviews published until 2017 [9] and three systematic reviews published posteriorly [10,11,12] underline as the main barriers the aspects related to: political and social context, such as lack of leadership and lack of coordination by the team; health organizational system context, such lack of staffing, and economic and time resources; clinical practice guideline context, such as recommendations that are not clear, and doubts about the quality of the evidence and rigidity; health professional context, such as lack of knowledge about the CPGs, negative attitude, and clinical inertia of practice and the belief that it is not part of one’s role; and patient context, such as lack of knowledge of the patients about the guidelines, the characteristics of the system and their illness, expectations that are contrary to the doctor’s, and lack of family support. The barriers highlighted in these systematic reviews are generally linked with the implementation of CPGs directed at interventions for the treatment of frequent pathologies and chronic diseases [10,11,12]. The studies on the implementation of CPGs of health promotion such as BF are less common, and have been mainly studied in environments of primary care and on aspects related to the prevention of chronic health problems and the implementation of practices related to lifestyle changes [13].

To date, studies related to the implementation of CPGs for BF have not been found. We only found a small number of studies that specifically studied the implementation of BFHI [14]. The barriers mentioned have some similarities with those specified in the literature about the implementation of CPGs mentioned in the previous paragraph, although with some their own specificities: involvement of the governments in the support of the BFHI, practices of the industry and legislation industry, lack of policies of BF support, contact routines with the mother and the newborn, the dependency of the hospital on infant formula companies, resistance in the promoting of BF due to the respect of the beliefs of the mothers, abuse of pacifiers, milk bottles, and scarce support from the families.

The BFHI shares certain similarities with the implementation of the CPG for BF; their objective is the support and promotion of BF in maternity and newborn services through the implementation and recommendations based on scientific and updated evidence, and they also use models that guide the process of implementation and evaluate specific result indicators. However, the implementation processes of the CPGs have specific particularities; they are generally part of the implementation programs that have a greater reach within the institution, use implementation processes that are specifically oriented to the implementation of evidence and the guides contain a greater number of recommendations. These differences justify the specific study of the barriers experienced by the managers and health professionals involved in the implementation. On the other hand, despite the current knowledge about the existence of barriers related with the implementation of the CPG, it is unknown until what point the barriers suggested are common to those linked to the implementation of a CPG on breastfeeding. Thus, the objective of the present study was to determine the barriers identified by the managers and health professionals involved in the implementation and sustainability of a CPG for Breastfeeding in a medium-sized hospital in Spain under the auspices of the Best Practice Spotlight Organization program.

## 2. Materials and Methods

### 2.1. Study Design

A qualitative approach was used, and an inductive thematic analysis [15] was performed to discover the experiences and expectations of the participants.

### 2.2. Settings and Participants

The study was conducted within the National Health System (Spain) in a 300-bed hospital. This center is not an accredited baby-friendly hospital. The Care Quality Department of the hospital prioritized the promotion of breastfeeding because the exclusive breastfeeding rate at discharge was only around 40%. In Spain, the rate of breastfeeding at 3 months is 72.4%. This hospital adhered to the Best Practice Spotlight Organization (BPSO^®^) Madrid, Spain, coordinated by the Nursing and Healthcare Research Unit of the Carlos III Health Institute (Investen-isciii), in collaboration with the Registered Nurses Association of Ontario (RNAO) [16]. Between 2012 and 2015, and following the BPSO^®^ program, five CPGs were implemented, one of them related to BF. The BPSO program includes multicomponent interventions for the implementation of the CPG. Among the different interventions, we find the training of the professional health workers, changes in the policies of the organization about breastfeeding, greater accessibility of the users, the creation of an exclusive breastfeeding room, etc. The implementation of the BF guide followed the conceptual framework adapted by the RNAO, the Knowledge to Action Framework (KTA), which is composed of six phases: (1) identification of the problem and (2) adaptations to the local context; (3) assessment of the facilitators and barriers; (4) adaptation and implementation of these interventions; (5) monitoring and evaluation of the results; and (6) sustainability. In 2015, the hospital was accredited as a BPSO^®^ hospital, and the sustainability phase began, which has been maintained until the present day.

The participants in the study were managers involved in the BPSO^®^ implementation program and health professionals from the maternity and pediatric units at the hospital where the CPG for BF was implemented, included health assistants, nurses, midwives, pediatricians, and gynecologists. A maximum variation sampling method [17] was used to obtain highly rich heterogeneous information with respect to gender, age, professional profile and role (manager or clinician). All the participants had been working in the health services where the CPG was implemented during the period of implementation and sustainability (2012–2017). The total number of professionals who worked in all the services was 82.

### 2.3. Data Collection

Semi-structured interviews were performed to obtain information, which were audio recorded and transcribed for their posterior analysis. The interviews took place between May and August 2017, and were given by the research team members (A.J.R.-M., M.R.-M., C.L.-C. and D.H.-A.), who had training and experience on interviewing. The participants who complied with the inclusion criteria were invited to participate, and a place, day, and time were agreed upon for the interviews. All the health professionals who were asked to participate accepted. A script (Table S1) with questions about the difficulties and barriers found in the implantation of the CPG was used, which asked for information from the most general to the more specific matters. A pilot study before the script was conducted as well [18]. The saturation criteria was applied to establish the number of informants needed [19].

### 2.4. Data Analysis

The sequence of 6 steps proposed for thematic analysis was used [20]: (1) Familiarizing yourself with your data; (2) Generating initial codes; (3) Searching for themes; (4) Reviewing themes; (5) Defining and naming themes; (6) Producing the report. All the authors participated in the thematic analysis and the interpretations of the results. The interviews were audio recorded, transcribed and imported to the qualitative research software MAXQDA version 12 (VERBI Software, 2015, Berlin, Germany) for analysis. The coding, themes and subthemes were agreed upon by the research team for their verification. When faced with discrepancies between researchers, these were resolved by consensus.

The study was reported according to the Consolidated Criteria for Reporting Qualitative Research (COREQ) [18].

### 2.5. Ethical Considerations

This research was approved by the research committee from the Health Area III from the Region of Murcia (ID: 002/2015). All the participants received an informational document about the purpose and process of research and participated voluntarily. They were allowed to ask and reflect before the interview, and their informed consent was obtained. Additionally, to ensure the confidentiality of the participants, the interviews were anonymized through the use of codes.

## 3. Results

A total of 20 health professionals participated, aged between 28 to 62 years old. The interviews lasted between 23 and 71 min. The characteristics of the participants are shown in Table 1.

Four major themes were identified: (1) Lack of resources and their adaptation; (2) Where, Who and How; (3) Dissemination and reach of the project to the professionals; (4) The mother and her surroundings. A detailed description of the themes and subthemes is found in Table 2.

### 3.1. Lack of Resources and Their Adaptation

One of the main barriers identified by all the health professional categories and profiles involved in the implementation of the CPGs were lack of resources. Thus, we find barriers related to the lack of resources in three levels: physical, economic and human.

#### 3.1.1. Physical Resources

Part of the period of implementation of the CPG coincided with an important architectural remodeling period of the pediatric unit and delivery room (always within the period of implementation and with a duration of 11 months). The professionals have an ambivalent discourse about this period. The midwives and nurses indicated that the renovations were an obstacle, but at the same time recognized that in the end, the implementation of the guide was favored due to the improvement of the spaces with respect to those that existed previously.

What happens is that in the provisional delivery room, well, we didn’t have space. E10.

The hospital is doing things. In pediatrics, a parent room was created during the renovation so that they could stay, so I don’t see physical barriers. E7.

Maybe the reforms have created them [limitations]. A lot of work has been done in the new structure. The division of the delivery room and maternity ward, having the resources dispersed too, could have slowed us down, so that we could not address the objectives well. E11.

The different categories and profiles manifested that the institution was not prepared at the level of the infrastructures to be able to provide high-quality care before the renovations.

*We did not count with many structures or many resources for doing it, we adapted to the structures, the resources, to what we had. E1.*

*On the one hand [barriers] that: the architectural barriers. E2.*

*Not having space, … E3.*

*Because if there had been a lactation room here in maternity, that would have helped a lot. An adapted room, where a woman could be with her child, that would help a lot. E4.*

#### 3.1.2. Economic Resources

A group of barriers identified by the interviewees was related with the lack of economic resources. It was also observed how these economic resources were identified as necessary during the implementation or maintenance period of the CPG and not only as a one-time intervention.

*Well, I don’t know if I should venture in saying economic, but due to the period we were going through, without assistance. E13.*

*I think that we were missing human, physical, and economic resources so that the guide becomes more important, or it’s going to stagnate. E4.*

Nevertheless, the managers believed that the personal interest was necessary, and that the implementation of a CPG implied savings for the organization. No clinician thought the same.

*I don’t think money is needed, what is needed is personal interest. E12.*

*Really, the BF guide has even meant savings. Or it means savings for the organization. E1.*

#### 3.1.3. Human Resources

The clinicians and managers, in all the professional profiles, manifested the lack of human resources. They indicated the existence of distancing between the efforts demanded from them and being able to do them, due to the lack of human resources.

*What happens? That there is also a human resources part. There is a lack of personnel. We are always lacking people. There is a lack of professionals. E6.*

*More personnel is needed, let’s leave it at that, I’m not going to specify categories. E16.*

The professionals indicated that there was a relationship between failure in breastfeeding and the insufficient number of professionals. They identified that the support of breastfeeding had specific events that could not be delayed, linked to each mother and newborn, and that their availability for tending to them was not always there due to the lack of personnel, especially when there was a smaller number of professionals (at night, weekends).

*… if we had more people for the weekend, perhaps. Because everything happens on the weekend, although you want to go a room, you can’t …, then this person is left hanging, without help, many who do not breastfeed well, the baby does not latch well, problems start to occur, and this makes difficult the follow-up later on. E4.*

*If you’re saturated with work, you cannot tend to the patient as you would like to. You are lacking human resources. E13.*

*[lack of] personnel, maybe it’s true, you start and make sure that the baby latches, but if you have four to five mothers, you are who you are, not more. You can’t spend more time with one and not tend to the others.*

Another situation that implies a barrier is hiring during vacation periods, with substitute professionals and their characteristics.

*Then summer comes, with the hiring, not too many substitutions. Then the demotivation starts. E6.*

*It is hard to motivate people who have not chosen to end up working in an area such as this, who are here not by choice but because he or she studied nursing. And the contracts are like that, they have ended up here …, then, motivating them is the hardest … I think they should be motivated in some way, making them understand that it is important because … that it’s one of their functions, just as in other floors they have other functions, here it is breastfeeding. E9.*

The lack of personnel is partly recognized by the managers, although at the same time they indicated that more personnel was available, and an investment had been made for this. Part of the managers, just as the clinicians, also recognized that if the professionals were asked to perform some interventions, then more resources were needed.

*If more personnel is solicited and they have not given it to you, it’s because they didn’t want to, I think, not because they could not. E1.*

*Yes, they have added midwives in the morning for the maternity ward […]. That has been done. E1.*

*But humans yes, hands are needed. And also, when the professionals ask, if they want us to do this, then they have to give us the other […]. And you know that the professionals were right and this tires you out. Giving an answer to the demands and you know that they are right. E1.*

#### 3.1.4. Lack of Time

The clinicians identified that the problem was the lack of time for performing interventions, and they linked it to lack of personnel. The managers think the opposite.

*I think the lack of personnel, but it’s the lack of time because there is no personnel. E15.*

*You need time. Time to sit with the mother, […]. The discharge we can do while on duty depends if there’s only one of us. If there’s only one, it could be that you can leave or not. We are lacking personnel. E3.*

*Time was available. It has been facilitated, there have not been problems of time or equipment. E11.*

#### 3.1.5. Adaptation of the Resources

On many occasions the resources were available, but their adaptation to the needs demanded by the implementation of the guide was necessary. Thus, we should distinguish the profiles of clinician and manager. The clinicians do not refer to this in a general manner; however, the managers attest that the process of data collection allowing posterior analysis has been complicated. This was pointed out as the starting problems of different degrees of implementation of the computer programs, different computer programs at different attendance levels, and bureaucratic problems faced to be able to obtain data that are adapted to the evaluation needs of the CPG.

*Well, it is a lot of work getting data from the computer program, especially because the services have their own clinical history … and we have had to ask the central services for authorization and help with getting these data out, as it was not possible through a normal exploration of data. E12.*

*The extraction of data has been difficult. Getting used to recording the information to be able to get it and evaluate these data. This has not only been hard, but it is still hard. Our computer system does not allow for a smooth extraction of data. Obtaining the data has been the hardest. E1.*

### 3.2. Where, Who and How

This theme includes different subthemes that are intimately related and linked to the place where the implementation takes place and the characteristic of those who do them, as well as the relationships between them. This is how the barriers are described that are related to context, the characteristics of the professionals themselves and their relationships.

#### 3.2.1. Work Context, Attitudes, Motivation and Preferences

There are peculiarities of the work context that entail certain barriers. The interviewees point out that there is no system of formal recognition for the quality of the work performed.

*Since it doesn’t matter who does more or who does less, it you do more, you receive the same recognition. E7.*

*Other barriers were the attitudes, motivation and preferences of the professionals.*

*Also our attitudes, this past years, I didn’t care much about breastfeeding. E17.*

*Yes, in the end it becomes something that you like, in the end it is a personal taste. E9.*

*I need motivation, otherwise this becomes very monotonous. E4.*

*Well, it depends on one’s interest, because there are people who don’t care about the subject of breastfeeding, or they don’t care about offering high quality care or anything else. I’m telling you like it is (laughs). E16.*

#### 3.2.2. We Have Always Done It This Way

Clinicians as well as managers have the opinion that there are people who do not update their knowledge and keep on performing their tasks as they did years ago. The history of this hospital is that is that for many years, the advantages of artificial milk were defended. They identified that it was difficult to achieve professionals changing their routines, and that some of these routines were perpetuated to younger professionals.

*There are people who say that the evidence, what’s that? We’ve always been doing this … nothing bad has ever happened, no child has died. E1.*

*That from one day to another they tell you that what you’ve done it’s not right and that now that’s the best thing, when our mothers gave it to us, because they didn’t have money, we were breastfed. Of course, it’s hard … E10.*

*We are still following erroneous health routines which makes so that the results do not change. Let’s see, some are young, but they are stuck with the old routines. There are a lot of young people who acquire the old routine from a colleague. And this is how it works, I don’t know, they question things. E20.*

*Well, if someone comes with ideas, that are not new, and that’s what it is [evidence], and they tell them “and now you come … after I’ve been here for 20 years and seen all I’ve seen … ”. I think there are people who have been with the subject of bottles for 30 years, and now it’s not easy for them. E6.*

#### 3.2.3. Always the Same: Breastfeeding

That breastfeeding plays such an important role is always pointed out by the managers as well as the clinicians as a barrier. They think that it is very repetitive, and that it has always had an important place in the training sessions.

*They are bored with the subject because they think of it as pounding information [on their heads]. They want to stress breastfeeding again, as if there was nothing else. E1.*

*Sometimes we say, come on…more breastfeeding courses, we already know everything! Sometimes we complain, I’ve had it up to here with BF already!!! E4.*

#### 3.2.4. Age

Among the personal characteristics, age has a very important role. The older professionals have a more relaxed attitude about BF. At the same time, their broad clinical experience is shaped by periods in which artificial breastfeeding “was beneficial”, and now they are living through a work period when natural breastfeeding is beneficial. Therefore, we are observing a closeness between scientific evidence and the young. They indicate that being older means less closeness with scientific evidence and vice versa.

*In this service, I think age has been an obstacle … Even when you have more experience and they can provide better advice because they have more life and professional experience … However, with the younger ones, they assume that when things come with evidence, they are good, you have to do them. E1.*

*Well, many times I didn’t care as much, because as I was saying, I’m older. E10.*

*[older professional] Let’s just say that I’m not very motivated, because it’s the end … you know? E10.*

*The interest about formula and its benefits had been promoted for many years. E11.*

#### 3.2.5. Inter-Professional Perceptions

The clinicians perceive the responsibility of breastfeeding differently. Most think that the protagonism of BF comes from the midwife. The managers have a two-sided view. On the one hand, they have the same opinion as the clinicians, and at the same time they think that the nurses have gained importance, as compared to the doctors, in terms of the implementation of the BF CPG.

*It is a multi-disciplinary guide. What happens with the nurses is that we didn’t have any importance in them. Or in this BF process, the importance was held by other professionals, gynecologists, and pediatricians. Everyone recognized a role in this process, including the patients. But the nurses, no […]. And the guide has given us a place. It tells us that we play a role and also an important role, they have to believe it and they have to assume it. E1.*

*Yes, they are not equal. I feel that the midwives and nurses, for example, are not equal. E1.*

*There are differences between services and between professional profiles. E20.*

*Between the specialized nurses, midwives and us in pediatrics with the doctors, [differences] will always be there.*

*Yes, of course, when there are midwives, the nurse calls the midwife from that floor so that she goes to the woman if she has a problem. But if the woman did not have any problem, you don’t have to wait for the midwife (she could be examining a woman or setting up a monitoring machine), you have to go. E2.*

*That this is not only about the midwife, and the nurse. E8.*

It is very striking and unanimous how they highlight, both clinicians and managers, the lack of involvement of pediatricians, affirming the lack of involvement of pediatrics as a discipline itself. However, the clinicians did not evaluate this to be innocuous. Rather, they identified that in some occasions, the actions of the pediatricians wasted all the work that had been done previously to promote BF when they made recommendations that did not take into account BF. The professionals indicated that the recommendations given by the pediatricians were very much taken into account by the mothers.

*Of course, it is just that sometimes, if they are not involved, if you have some pediatricians and some gynecologists who are not involved with breastfeeding, it is a giant obstacle, because one word, one recommendation from them and all the work you have done before falls apart. E9.*

*No matter if nursing and nursing assistants know something, if a pediatrician comes and gives completely opposite advice, what are you going to do? Are you going to discuss it in front of the woman? No. And who is the woman going to listen to? To the pediatrician, of course … E6.*

*They, on the subject of breastfeeding …, the pediatricians do not become involved too much. E19.*

In general, the doctor profiles related with the promotion of BF, and to whom the CPG was directed, where not involved enough. This was even identified by the doctors themselves.

*Perhaps we the pediatricians are invited to the breastfeeding courses, but we ignore them and many times we don’t read the posters. They are for everyone, but we exclude ourselves. E13.*

*The doctors, not at all. The doctors in this project are not involved. I don’t know what else you want. They have not wanted to become involved, they have thought of it as something for nursing exclusively. The pediatricians do not see that BF saves lives and the gynecologists I can’t even tell you. E20.*

*Well, I can tell you that in the last BF training course, as far as I know only one went from the entire pediatrics staff, and he is a resident. E13.*

The figure of the midwife represents a barrier when present, as the other professionals identified her presence with the automatic assigning of tasks related to BF and the exception of having to perform these types of activities.

*Here in the maternity ward, since the figure of the midwife it’s here, it makes it so that BF is not linked to nursing, because it makes it so that nursing [department] relaxes about this aspect, they say, no, no, no, the thing is that the midwife is here and she is in charge of the breastfeeding subject. E16.*

*It is a subject that is left for the midwives because they have more involvement in the subject. […]. E3.*

BF is not thought of as a clinical activity by the professionals.

*I think so, there is not much involvement by the personnel. And another thing is that the subject of breastfeeding is difficult to place, it’s not part of the tasks of the maternal-pediatric wards. E6.*

*You do what you have to do, what is really important for us, and the breastfeeding, that, that … it is indispensable, but … E3.*

*That breastfeeding is not for them [the doctors]. But anyway, they are more centered on the clinic, in the pathology part. Although the births are not pathologies … but for them, this like, I think that it is a small matter, which does not require their intervention. E9.*

### 3.3. Dissemination and Reach of the Project to the Professionals

#### 3.3.1. Dissemination

Managers and clinicians affirm that the dissemination of the project to the professionals should be improved. The managers refer to this aspect as something that is very complex, and although they have been working on this for a long time, good results have not been achieved. Some of the clinicians indicated that the board of directors should have presented this implementation to the hospital services.

*It was slowly done, I think that there’s people who do not know that it has been done. Maybe they have wanted to do it that way and not garner too much attention. E1.*

*When we have the training of promotors [of the CPG], we also say that channels should be created, but I see that it is still something that is difficult and that we are still not doing it well. […]. We do not establish this communication channels well. Then, there is a lack of information for the people. The information does not arrive to them. And look we have been doing this for years, but the mechanisms for transferring the information are still needed. E1.*

*As for implementation, I think that it should have been done with a drum and snare, with a music band if possible. The thing is that there are people at the hospital who do not know that a CPG is, that it’s a protocol, there are people who do not … E2.*

*First, I think that the management should have presented the services, saying that this is a project, that it is not a few crazy people who want to foment BF. E6.*

#### 3.3.2. Reach

The managers identified that, although all the areas covered by the hospital received the same information, training, and resources, the implementation of the CPG was unequal. They (managers and clinicians) identified that the preparation reached by the mothers before the birth was dependent on certain factors of primary care where the CPGs had been implemented efficiently.

*Because with primary care, well, we have worked with it, and we have not been able to expand it to other primary attention centers. E11.*

*A form was made, a sheet that explained that a visit to primary care should be done […] that the midwife had to speak to her about BF. Then the midwives had to complete and save it in the mother’s book, and the only ones that I see are the ones sent by the midwives from the [blinded] area, and it is precisely the place where the BF support group that works is located, the rest, not. E6.*

### 3.4. The Mother and Her Surroundings

The health professionals identified some barriers related to the mothers. On the one hand, they identified the lack of preparation in the habitual care contexts prior to the birth, referring to the preparation in primary care. On the other hand, they identified barriers related with the influence of close family members, as well as the prior decision about breastfeeding before going to the hospital to give birth. Lastly, the BF culture of the mother was also identified.

#### 3.4.1. Lack of Preparation before the Birth

Clinicians as well as managers also indicated that the pregnant women did not have enough preparation from primary care related to BF. They even thought that counting those with supportive partners could be interesting. They think that this is essential for the good functioning of the CPG.

*It is important from the health centers, mother’s education, and that the mother has a good base. E14.*

*Well, I don’t know, but maybe the fathers should also be taught something, maybe some things or some hints so that they also know … E15.*

*Like in many things, it depends on the health professional you get … There are other health areas that have not thought about it. E9.*

*But so that it is clear for the mother, I think that in primary care, the midwives, pediatricians and the rest have to make it clear for the mother. Then, if you don’t work on it from the beginning, then in the end we won’t have good performance. E10.*

*I think the mothers need more information. I think they don’t come to give birth with information about breastfeeding, they have many doubts and this is a hindrance when we try to help them … E19.*

#### 3.4.2. Close Family Members and Prior Decisions

The professionals indicated the influence of the family on BF was a strong negative factor. Sometimes, the grandmothers were identified as being especially negative. The professionals had an ambivalent discourse, where it was observed that the family also supported BF, but pointed out that in many occasions it was a clear inconvenience. They indicated that during the moments of implementation of breastfeeding they were critical and very fragile, and the mothers were heavily influenced by the opinions from their surroundings. These influences determined the absence of BF on many occasions. They identified that the “open door” policy (access during the entire day to the hospital rooms) was negative for BF, because spaces for intimacy were not created and there were always visitors in the rooms.

*Overall, the role of mothers-in-law and grandmothers, has a strong influence. This makes it difficult for the personnel and being able to provide advice or give this recommendation, because the family has a strong influence. E1.*

*Because the greatest barrier that we have now are the family members. E2.*

*Also the information that she had received from her family. Here we are fighting to follow the guide, you understand? But sometimes the parents come with demands, you know, that can’t really tell them … E14.*

*To top it all, here comes the grandmother and sticks a bottle to the baby, dear lord. E8.*

*Well, I think that sometimes the family is a negative factor. E16.*

*BF is a very fragile subject. The thing is, any person can have an influence, we say this even of the family members, and here sometimes we find the family members, and they obstruct many times, sometimes they help and other times not, many times, unfortunately, they don’t help. E9.*

The clinicians also identified that the cultural and social aspects also had an influence, in that BF was not in vogue, and that women come here with the idea of not BF.

*Many times they come here with the pre-conceived idea that they are not going to BF. E13.*

*You ask around, without pressuring them, because many of them tend to have the decision made. E3.*

*Because BF, the mother is the first one who should want that. E18.*

#### 3.4.3. Transversality

Two transversal themes appear that have an influence on the mothers. On the one hand, the socialization of BF and the value it has on society (including health professionals), and on the other hand, the presence of the variable immigration of mothers.

Managers and clinicians confirmed the lack of social awareness on the subject of BF. They pointed out that this was the case at the level of the population, as well as for health professionals themselves. They point out that not working on this socialization is a mistake.

*In Spain, for whatever reason, we don’t have this social culture of BF. And it’s very hard to break this lack of preparation of many professionals, the families, etc … The problem we have here is the low cultural level associated with the Spanish people. Not the low cultural level of the immigrants who by default will BF due to culture. It is the Spanish woman who is not prepared, who will not breastfeed, and what we find 80% of the time. E20.*

*I think once the awareness of the entire world changes in general, it will be easier. It’s just that were are in the hardest part, I think. Right now were are in the hardest point. E18.*

The immigrant mothers have a series of peculiarities that are identified as barriers: mainly language and culture. Difficulties were also identified in the access to health services, for example, the mother education classes.

*Then the language barrier. […] It’s difficult to talk about breastfeeding when you don’t understand the basics, then it’s very hard to inform this person, then, if it’s obvious, it’s obvious. E20.*

*Moroccans …. because, of course, they don’t understand because of the language, even if they go to the classes … (I don’t know if they go to motherhood education or not). E4.*

*From South America, we have more Ecuadorians, and the north part of Morocco. They even buy baby bottles. E19.*

## 4. Discussion

The results of our study provide updated and novel knowledge of the barriers perceived by all of the professionals involved in the implementation and sustainability of a CPG for BF in a hospital context. New empirical results are provided about a subject that has not been studied from a multidisciplinary perspective, with the inclusion of managers, with a CPG for health promotion, and temporarily framed within the BPSO^®^ program of CPGs implementation.

We found similarities with the barriers that had previously been found in research studies related to the barriers to the implementation of CPGs, and more specifically to the implementation of the BFHI [10,11,12,14]. In a prominent manner, and above the other barriers at the level of the health organizational system context, the lack of human, physical and time resources was highlighted. These relate the failure of BF to the scarcity of interventions that could be performed due to the lack of personnel, results which coincide with many systematic reviews showing the necessity of effective professional support for BF to be prolonged [21,22,23]. The literature indicates that to improve this practice, it could be necessary to provide more resources for its implementation, but this may not be a sufficient step in isolation [12]. Additionally, the lack of time was constantly mentioned in the clinician’s discourses. This coincides with other studies, which point to the lack of time as a barrier commonly mentioned by health professionals in most research studies [24,25,26,27]. Another aspect pointed out as a barrier is the constant rotation of professionals, which makes the implementation and maintenance of the CPGs difficult [28]. As shown in previous studies, a stable and good-sized professional staff is indispensable for the implementation and promotion of evidence-based practice (EBP) in any hospital organization [29].

The managers, although they agreed with the clinicians about the need for more health professionals, and although they recognized that this was the reason more personnel were hired, disagreed that they did not have enough time to provide recommendations, and argued that this was a matter of personal interest. As for the resources, the managers also reported that the monitoring and exploitation of the data was difficult to adapt to the needs of the evaluation of the guide. Many studies have pointed out the importance of this in terms of improving the collection of data for the purpose of their critical analysis [30,31].

Many aspects related to the health professional context have been shown to be barriers for the implementation of the BF guide. The lack of attitude and motivation among health professionals with respect to the subject of breastfeeding has been pointed out in particular, indicating that the work performed was not recognized. The lack of motivation and the negative attitudes towards certain interventions and the lack of professional recognition was related to the lack of compliance of the recommendations of the CPG [11,28,32]. Additionally, aspects related to inter-professional perceptions had an important presence among the barriers in the health professional context. alit is known that all of the stakeholders must participate in the adaptation and implementation to ensure that its needs are satisfied [33,34,35,36], and this is facilitated by the process of application of the guide. However, above all, it is the figure of the midwife that was highlighted by all the professionals. In part, the occurrence of this is reasonable, as the professionals who apply the EBP are found in specific contexts [37], or are better trained [38,39], and are therefore the most adequate for promoting the development and implementation of the CPG [34,36]. Nevertheless, in our research, the role of the midwife was ambivalent. Despite being key promoters, they were also indicated as barriers for the implementation of the CPG. It is possible that the presence of the midwife, or even her absence, created a feeling that they were the ones who had to do these interventions, creating resistances or doubts towards applying the interventions. In international contexts, where the figure of the lactation consultant has been promoted as the expert on BF support, it has been described that in some centers, the commitment of professionals decreases when counting on a figure who is an expert on breastfeeding, and that even the mothers only wanted to be cared for by these professionals [40].

There is also a perception transmitted by all the clinical profiles and managers regarding the insufficient participation of the doctors in the implementation of the CPG. There are research studies that coincide with this finding, and they relate this to a lack of incentives [41], the feeling of loss of control of their function, or the obligation to make important changes [11,42]. The lack of knowledge and training on breastfeeding has also been pointed out [43]. We also interpreted it in the sense that BF support comprises interventions for the promotion of health. Previous studies have indicated the existence of distancing of the professionals from activities that are not purely clinical [44,45]. This, as well, has negative repercussions on the rest of the professionals, as they suggest that not only did they not collaborate, but that this caused the work of others to go to waste [43]. In this sense, achieving the collaboration of other clinicians implied in the implementation of a guide is a key factor for good functioning.

The barriers mentioned to a lesser degree were linked to the perception that BF was a priority, and was a repetitive subject in training activities. The overload of information in training activities for health professionals has been described as a barrier [46]. Likewise, age and resistance to change have been shown to be conditioning factors. In line with other studies, the older professionals felt less involved in the application of the recommendations of the BFHI [40], and the older workers were also more resistant to change [14].

The clinical practice guideline context refers to barriers related to the perception of the guide and the process of implementation. In this case, the existence of preferences among the professionals was perceived to be a barrier, as they were perceived to be more effective than the recommendations presented by the guide itself, an aspect that has been frequently mentioned in other studies [47]. However, difficulties related to the complexity of the activities presented by the guide were not mentioned, in contrast to the findings in other studies [37]. This is perhaps due to this CPG not describing complex interventions, and the use of complex technology was not necessary; therefore, the professionals are able to acquire the knowledge more rapidly, feeling more secure when performing the interventions proposed by the CPG. Research has been reported that indicates that simple and accessible interventions have a greater probability of being applied [48], although this hypothesis should be validated in future research studies.

There was a small number of barriers related to the political and social context. The clinicians described not knowing in depth what the project for the implementation of the CPG consisted of, and indicated that a good dissemination of information had not been carried out. In this sense, many studies have underlined the importance of dissemination through formal and informal networks so that the participation of a greater number of professionals is facilitated [49]. Additionally, it has been shown that greater efforts should be made to expand the reach of the guide to primary care. Studies on the barriers for BFHI mention the lack of integration of health services, which translates into poor communication between facilities, limiting the support from primary care to the mothers [14].

The barriers related to the patient context referred to the lack of pre-natal preparation of pregnant women with respect to BF, an aspect that, according to what has been documented, contributes to the low motivation for starting BF, or to its early abandonment [50]. The professionals identified the influence of the family as a barrier, along with cultural and social aspects. These coinciding results show that the support of the family and the companionship of the partner are key determinants for the success or failure in the practice of BF [51]. The beliefs of the mothers and the families with respect to cultural aspects, which may even vary based on the woman’s country of origin, implies an added difficulty that has been described previously [40].

The findings presented could be of help for researchers, clinicians and managers interested in the implementation of BF guides. It is important to point out that in order to achieve the correct implementation of the CPG, it is essential to take into account the social reality and the context in which these will be applied [52]. For example, the barriers described in other studies [14,53] with respect to the influence of the formula industry and the lack of support from the organization, were not described as a barrier for the implementation in our study. On the other hand, phase 3 of the BPSO implementation program for the guides indicates that an evaluation of the barriers and facilitators should be made before designing implementation strategies. The findings show us that barriers change throughout the process of implementation. Thus, the lack of infrastructure, which was considered a barrier at the start of implementation, became a strength afterwards. Likewise, new barriers appeared with the application of strategies for the implementation of the recommendations that were impossible to detect initially. It would be interesting for the model proposed by the BPSO to incorporate periodic analyses of barriers, facilitating their analysis and the discussion with the professionals, so that corrective measures could be proposed for the adaptation to the local reality and context.

### Limitations

The extrapolation of results to other contexts should be made with caution. On the one hand, the qualitative methodology has a low external validity, and the results themselves are also strongly dependent on the local context. Another limitation is that some participants knew that part of the research team participated in the implementation project, and this could have influenced the information provided.

## 5. Conclusions

This research identifies the barriers perceived by the professionals involved in the implementation of the CPG by incorporating the point of view of the managers. Novel barriers appeared, such as the ambivalent character of the role of midwives, and the fact that the CPG for BF was related to the promotion of health. The efforts to promote the program of implementation should be continuous, addressing and planning different dissemination strategies, and the expansion of primary care services should be prioritized. The most important issue from the point of view of the management of services is to work on the adaptation of the computer systems for recording the key indicators of the guide in order to improve the updating of results and to offer feedback to health professionals on the progress of implementation. More efforts should be made to delve into the interpretations of beliefs and behaviors of mothers and families in their social contexts. Lastly, a greater involvement and collaboration of all the professionals with respect to BF is required, especially doctors. Midwives and nurses, in close collaboration with managers, should lead this initiative.

## Figures and Tables

**Table 1:** Characteristics of the participants and duration of the interviews.

Participants *	Gender	Professional Profile	Age	Years of Professional Practice	Duration of Interviews (Minutes)
20	2 Men 18 Women	Hospital Administration: 3 Midwife: 5 Pediatric nurse: 3 Pediatrician: 1 Maternity Nurse: 3 Gynecologist: 1 Pediatric healthcare assistant: 1 Maternity healthcare assistant: 1 Delivery room healthcare assistant: 1	28–62	4–40	23–71

* To guarantee anonymity, the characteristics of each participant are not identified individually.

**Table 2:** Themes and subthemes.

Themes	Subthemes
Lack of resources and their adaptation	Physical resources
	Economic resources
	Human resources
	Lack of time
	Adaptation of the resources
Where, Who and How	Work context, attitudes, motivation and preferences
	We have always done it like this.
	Always the same thing: breastfeeding.
	Age
	Inter-professional perceptions
Dissemination and reach of the project to the professionals	Dissemination
	Reach
The mother and her surroundings	Lack of preparation before the birth
	Close family and prior decisions
	Transversality: BF and it socialization, and immigration

## Supplementary Materials

The following are available online at https://www.mdpi.com/1660-4601/17/17/6248/s1. Table S1: Semi-structured interview script.
