# Supplementary material for: Barriers Perceived by Managers and Clinical Professionals Related to the Implementation of Clinical Practice Guidelines for Breastfeeding through the Best Practice Spotlight Organization Program
Source: Int J Environ Res Public Health. 2020 Aug 27;17(17):6248. doi: 10.3390/ijerph17176248 (PMC7504213; doi:10.3390/ijerph17176248)
Supplement: Supplementary file 1 [file ijerph-17-06248-s001.pdf]

Table S1. Semi-structured interview script.

1.	What do you know about the project to implement the breastfeeding guide? How would you improve the accessibility of the GPC? Do you think the implementation of this guide is a priority, if not, which do you think is a priority?
2.	Do you think that CPG has the best available evidence for its patients? What is your view on the changes that have taken place to implement this guide? Do you think CPG is useful? If so, which particular part do you find unhelpful? If not, why not? Do you think that the implementation of the guide has changed the way you work? What things are missing during the process of implementing the breastfeeding guide that would have made it easier for you to adhere to the recommendations?
3.	How has the implementation of the GBP influenced the outcomes of patients and other related individuals? Are the recommendations being used? Is the implementation initiative working? Are the recommendations clear and easy to understand?
4.	In your opinion, what would be the most effective way to improve the use of the guideline recommendations? Do you think breastfeeding CPG is necessary to improve clinical practice? Is it feasible to implement it in your unit?
5.	Do you know what some of the guideline implementation strategies are? Which one is most appropriate for the selected GBP
6.	Do you conduct your practice in accordance with CPG? What are the barriers or reasons for using or not using CPG? Are there any barriers to implementation of recommendations? Are there budget issues? Staff competency issues? Leadership issues? Workload issues? Cultural and attitudinal issues? Time to evaluate results? Do you think there are sufficient human and financial resources, time and space? Does implementation increase or decrease workload?
7.	Does the top management openly support the implementation? If not, how can this support be achieved?
8.	What kind of recommendations have been most easily followed? Why do you think this has happened?
9.	How does your clinical experience fit in with the recommendations?
10.	Are the patient's or family's preferences taken into account?
